# Secreted protein markers in oral squamous cell carcinoma (OSCC)

**DOI:** 10.1186/s12014-022-09341-5

**Published:** 2022-02-08

**Authors:** Madiha Mumtaz, Irene V. Bijnsdorp, Franziska Böttger, Sander R. Piersma, Thang V. Pham, Samiullah Mumtaz, Ruud H. Brakenhoff, M. Waheed Akhtar, Connie R. Jimenez

**Affiliations:** 1 School of Biological Sciences, University of the Punjab, Lahore, 54590 Pakistan; 2 Department of Medical Oncology, Cancer Center Amsterdam, OncoProteomics Laboratory, Amsterdam UMC, VU University Medical Center, De Boelelaan 1117, 1081 HV, Amsterdam, The Netherlands; 3 Shalamar Medical and Dental College, Lahore, Pakistan; 4 Department of Otolaryngology/Head and Neck Surgery, Cancer Center Amsterdam, Amsterdam UMC, Vrije Universiteit Amsterdam, Amsterdam, The Netherlands; 5 Department of Urology, Cancer Center Amsterdam, Amsterdam UMC, de Boelelaan 1117, 1081 HV, Amsterdam, The Netherlands

**Keywords:** Proteomic quantification, HNSCC, Secretome, Oral squamous cell carcinoma, Public biofluid datasets, Human salivary proteome

## Abstract

**Background:**

Oral squamous cell carcinoma (OSCC) is a main cause of oral cancer mortality and morbidity in central south Asia. To improve the clinical outcome of OSCC patients, detection markers are needed, which are preferably non-invasive and thus independent of a tissue biopsy.

**Methods:**

In the present study, we aimed to identify robust candidate protein biomarkers for non-invasive OSCC diagnosis. To this end, we measured the global protein profiles of OSCC tissue lysates to matched normal adjacent mucosa samples (n = 14) and the secretomes of nine HNSCC cell lines using LC–MS/MS-based proteomics.

**Results:**

A total of 5123 tissue proteins were identified, of which 205 were robustly up- regulated (p-value < 0.01, fold change > + 2) in OSCC-tissues compared to normal adjacent tissues. The biological process “Secretion” was highly enriched in this set of proteins. Other upregulated biological pathways included “Unfolded Protein Response”, “Spliceosomal complex assembly”, “Protein localization to endosome” and “Interferon Gamma Response”. Transcription factor analysis implicated Creb3L1, ESRRA, YY, ELF2, STAT1 and XBP as potential regulators. Of the 205 upregulated tissue proteins, 132 were identified in the cancer cell line secretomes, underscoring their potential use as non-invasive biofluid markers. To further prioritize our candidate markers for non-invasive OSCC detection, we integrated our data with public biofluid datasets including OSCC saliva, yielding 25 candidate markers for further study.

**Conclusions:**

We identified several key proteins and processes that are associated with OSCC tissues, underscoring the importance of altered secretion. Cancer-associated OSCC secretome proteins present in saliva have potential to be used as novel non-invasive biomarkers for the diagnosis of OSCC.

**Graphical Abstract:**

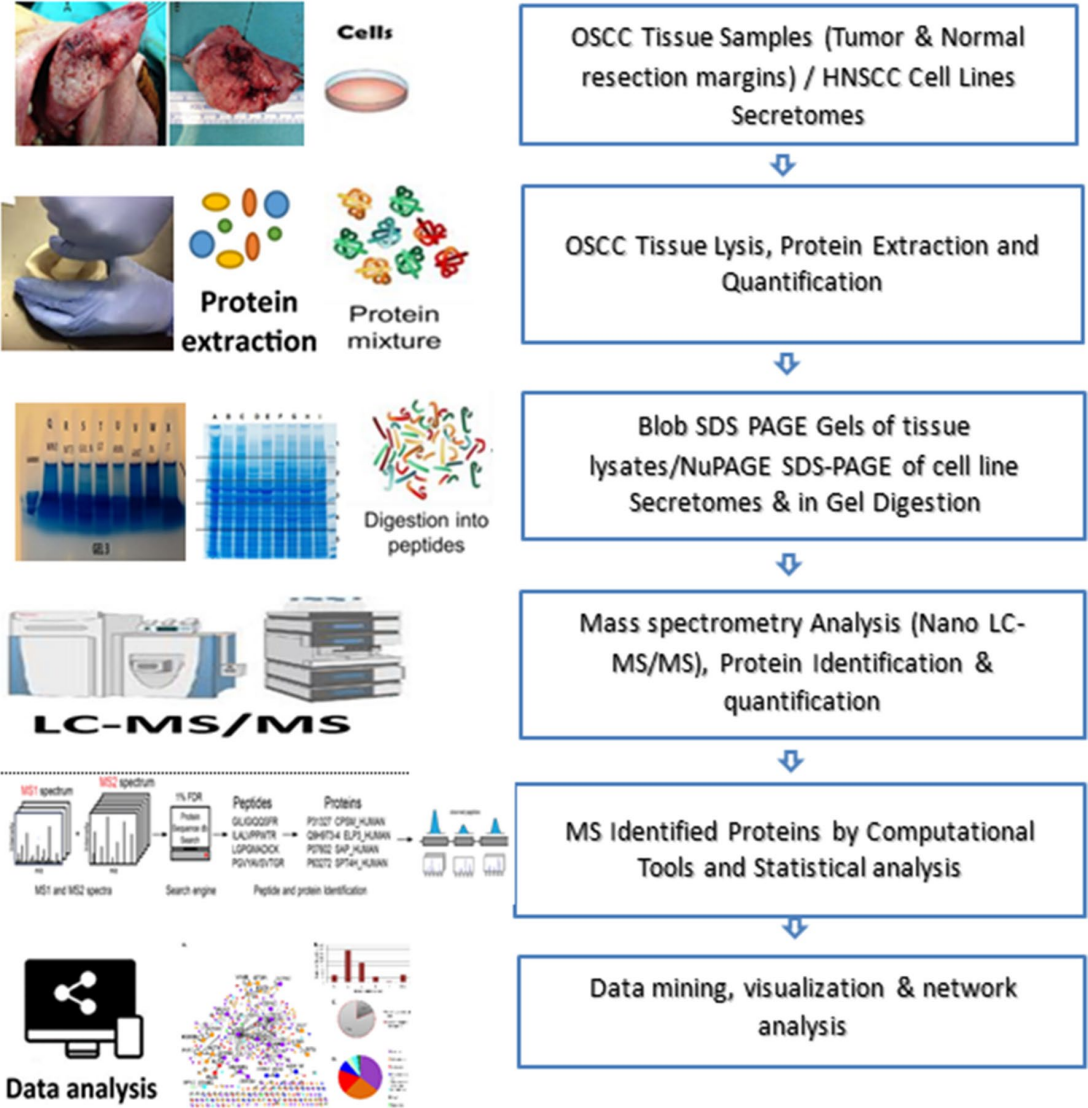

**Supplementary Information:**

The online version contains supplementary material available at 10.1186/s12014-022-09341-5.

## Background

Head and neck squamous cell carcinoma (HNSCC) arises from the mucosal epithelium of the oral cavity, nasopharynx, oropharynx, hypopharynx (and larynx. Squamous cell carcinoma is the most common histological type of head and neck cancer, accounting for 90% of all head and neck malignancies. Oral squamous cell carcinoma (OSCC) and oropharyngeal squamous cell carcinoma (OPSCC) are the most common HNSCC [1]. OSCC is the 6th most common epithelial malignancy world-wide and associated with high morbidity and mortality. Therefore, OSCC is a global growing concern for public health [2].

The main risk factors related to HNSCC development are use of smokeless tobacco products, chewing/smoking tobacco, genetic predisposition, alcohol consumption and/or infections with human papillomavirus (HPV). In Pakistan, Taiwan, India and China several social behaviors, such as chewing raw tobacco, gutka, paan (betel quid), chaliya (areca nut), manpuri and niswar are among common addictions [3]. Initial symptoms are often mild, causing a delay in presentation. Diagnostic care includes clinical examination of the oral mucosa and histological evaluation of tissue biopsies. However, these methods generally lead to a late diagnosis of OSCC. Therefore it is important to develop new non-invasive diagnostic tools that can detect the presence of OSCC at an earlier stage preferably in saliva or oral rinses.

To identify new biomarkers that can improve the diagnosis of OSCC, analysis of global protein expression and secretion using mass spectrometry has gained increasing interest [4]. Global identification of altered expression of proteins in OSCC tissues may provide an unbiased approach to find vital biological pathways, leading to improved understanding of the primary molecular mechanism responsible for disease development and progression. Non-invasive markers may be best identified using biofluid analysis, yet plasma proteomics has been hampered, mainly because of the vast complexity and great dynamic range of proteins in blood. To overcome this challenge, cancer cell and tumor tissue secretome analysis have emerged as alternative approaches to identify candidate tumor serological markers [5, 6].

Recently, the power of clinical proteomics was shown in a large scale analysis of 66 HNSCC tumors [7]. Comparison to paired normal adjacent tissues revealed profound alterations in multiple biological processes such as downregulation of platelet degranulation, acute inflammatory response, fatty acid metabolic process and muscle system process and upregulation of protein hydroxylation, leukocyte migration, cell chemotaxis, and angiogenesis [7].

In this study, we set out to identify “non-invasive” protein biomarker candidates that can be detected in oral cancer-relevant biofluids. To this end, we employed mass spectrometry-based proteomics, to examine protein expression profiles in OSCC and adjacent normal tissue of 14 patients along with analysis of secretomes of nine HNSCC cell lines. To further prioritize our candidate markers for non-invasive OSCC detection, we integrated our data with public biofluid datasets: salivary proteome healthy vs. OSCC dataset by Chu et al. [8], Human salivary proteome by Sivadasan et al. [9] and available Normal Saliva Proteome database (https://salivaryproteome.nidcr.nih.gov/). Moreover, we also describe oral cancer associated biological pathways and TFs involved in regulating those proteins to gain understandings of differential biology of secretion.

## Materials and methods

### Collection of tissue specimen of OSCC patients

This study includes 14 patients; Tissue samples of male and female subjects diagnosed with oral squamous cell carcinoma (OSCC) were collected from local Hospital Lahore, Pakistan according to the approved guidelines of the Ethical Review Committee of the King Edward Medical University [Ref. No. 306/RC/KEMU] with consent of the patients. Patients with either Hyperplasia, Papilloma, Pleomorphic Adenoma, Soft Tissue Tumor, Odontogenic tumors, HIV infection, hepatitis C, B and/or with history of current or earlier chemotherapy or radiotherapy were excluded from the study. All tumors of OSCC patients included in the study were HPV negative. Tumors were TNM staged according to AJCC recommendations. After surgery tissue specimens (tumor and adjacent non-tumor tissue) were immediately frozen in liquid nitrogen and stored at − 80 °C for future use. Non-tumor tissues were cancer cell-free and around 80% cancer cells were present in stored tumor tissues as revealed by pathological evaluation. The histology information showing cases of moderately differentiated SCC and well-differentiated SCC in (Additional file 1: Fig. S1). The tumor composition consisted of ~ 80% cancerous and 20% non-cancerous cell presence ensures a high likelihood of selecting proteins originating from cancer cells. To further confirm this, we generated secretome data from a panel of cancer cell lines and focused our attention on the overlapping proteins. Clinical information of patients was collected from the hospital files including medical history and demographic data (Table 1).

**Table 1:** Clinical information of the study subjects

Sr. No	OSCC patients (Gel labels)	Sex	Age	Family history	Clinical presentation	Tumor localization	Histological diagnosis*	Staging TNM	Smoking/Tobacco	Pan, Gutka, Chalia Chewer	Alcohol consumption	Scioeconomics status^¥^
1	ST1	M	70	No	Lesions with raised exophytic margin	Buccal Mucosa	3	Stage IVA (T4aN1M0)	Smoking	None	Yes	2
2	MT1	M	62	No	Ulcerative type lesion	Tongue	3	Stage IVA (T4aN1M0)	None	Gutka	Yes	2
3	ET	M	60	No	Ulcerative type lesion	Tongue	1	Stage II (T2N0M0)	Smoking	None	No	1
4	ST2	M	55	No	Lump	Buccal Mucosa	2	Stage III (T2N1M0)	Smoking	None	No	1
5	MT2	F	40	No	Ulcerative type lesion	Molar area (Mandibular Retromolar Area)	3	Stage IVA (T4aN0M0)	None	Pan & Chalia	Yes	1
6	NT	M	51	No	Lichen planus	Buccal Mucosa	2	Stage IVA (T4aN0M0)	None	Paan	No	1
7	BT	M	38	No	Ulcerative type lesion	Tongue	3	Stage IVA (T4aN0M0)	Smoking	None	No	3
8	AT	M	45	No	Lichen planus	Buccal Mucosa	3	Stage IVA (T4aN1M0)	Smoking	Pan & Gutka	No	1
9	MT3	F	65	No	Ulcerative type lesion	Hard Palate	3	Stage III (T3N0MX)	None	Pan & Gutka	No	1
10	GT	M	60	No	Ulcerative type lesion	Soft Palate	3	Stage III (T2N1M0)	Smoking	None	Yes	3
11	ANT	M	36	Yes (Father)	Ulcerative type lesion	Molar area (Mandibular Retromolar Area)	2	Stage IVA (T4aN2M0)	Tobacco Chewing	None	No	2
12	JT	M	35	No	Mixed ulcerative & exophytic lesion	Buccal Mucosa	3	Stage IVA (T2N2M0)	Smoking	Chalia	No	1
13	GT	F	30	Yes (Mother)	Lesions with raised exophytic margin	Tongue	2	Stage III (T3N0M0)	None	Chalia	No	1
14	NOT	F	55	No	Verrucous type lesion	Lip	3	Stage IVA (T4aN0M0)	Smoking	None	No	1

*1. Poorly Differentiated SCC, 2. Moderately Differentiated SCC, 3. Well Differentiated SCC. ^¥^1. Low scioeconomics status, 2. Middle scioeconomics status, 3. Upper scioeconomics status

### Tissue lysis and protein extraction

As mentioned previously [10], 200 mg of the tumor tissue and control tissue samples were homogenized with liquid nitrogen in a pestle with mortar, suspended in modified chilled lysis buffer, vortexed for 1 h and centrifuged for 90 min at 1400 rpm at 4 °C (Eppendorf Centrifuge, 5417R). The resultant supernatant was aliquoted and stored at − 80 °C. Bradford assay [11] with bovine serum albumin (BSA) as reference was used for estimating protein concentration in tissue lysates.

### Cell line cultures

Dulbecco’s modified Eagle’s medium (DMEM) containing 5% fetal calf serum (FCS), 2 mM L-glutamine and antibiotics was used to culture all cell lines (Table 2) used for this study [12]. Cultures were maintained with 5% CO_2_ at 37 °C in a humidified atmosphere. HNSCC cell lines UM-SCC-22A, UM-SCC-6, UM-SCC-11B were obtained from Thomas Carey (University of Michigan, USA). FaDu cells were purchased from ATCC [13] and cell lines VU-SCC-120, VU-SCC-1131, VU-SCC-096, VU-SCC-040 have been previously established at Amsterdam UMC location VUmc, The Netherlands [14, 15]. In addition, all cell lines were tested frequently for mycoplasma (Mycoalert, Lonza, Verviers, Belgium). Cell lines were authenticated by visual inspection, *TP53* mutation sequencing, and PCR profiling. Through GP5 + /6 + DNA PCR the status of HPV was confirmed [16]. All cell lines, we used were HPV- negative.

**Table 2:** Panel of HNSCC cell lines

Human HNSCC cell lines		Gender	Stage	Primary tumor site	HPV status
HPV-NEG HNSCC	VU-SCC-040^a^	Female	T3N0	Tongue	Negative
	VU-SCC-11B^‡^	Male	T2N2a	Supraglottic larynx	Negative
	UM-SCC-22A	Female	T2N1	Hypopharynx	Negative
	UM-SCC-6	Male	T2N0	Base of tongue	Negative
	VU-SCC-120	Female	T3N1	Tongue	Negative
	VU-SCC-096	Male	T4N1	Retromolar trigone	Negative
	FaDu^b^	Male	NA	Hypopharynx	Negative
FA-HNSCC	VU-SCC-1604	Female	NA	Tongue	Negative
	VU-SCC-1131^‡^	Female	T4N2b	Floor of mouth	Negative

*NA* not annotated. ^a^TP53 wild-type cell line. ^b^Cell line from a sporadic HNSCC tumor with a de novo Fanconi gene mutation [47]. ^**‡**^Local recurrences of primary tumo

### Cell line secretome collection

Conditioned medium from cell lines was collected and processed as described before [17]. Briefly, cells were seeded in T175 flasks and cultured until ~ 70% confluence. Cells were subsequently washed three times with PBS and incubated for 24 h in serum-free medium prior to harvest of the conditioned medium. Following centrifugation (5 min at 500 × *g*) and passing of the supernatant over a 0.45 μm filter for removal of cell debris, the medium was concentrated to ~ 200 μl using an Amicon 3 kDa filter (Merck Millipore). Protein concentration determination was performed using BCA Protein Assay Kit (Pierce, Thermo Scientific). Following addition of LDS Sample Buffer (Invitrogen) containing 100 mM dithiothreitol, 30 µg of secreted protein lysates were separated using gel electrophoresis.

### Protein gel electrophoresis of tissue lysates and cell line secretomes and in-gel digestion

Prior to mass-spectrometry analysis, proteins were fractionated by gel electrophoresis followed by coomassie blue staining. Gel electrophoresis and in-gel digestion were done as described earlier [17]. Tissue lysates (25 µl) were separated on a 12% SDS-PAGE acrylamide gel (BioRad, Hercules, CA), at 100 V until the sample had entered just into the running gel. The cell line secretomes were separated until the bromophenol-blue dye-front reached the end of the running gel. Following electrophoresis, gels were fixed in 3% phosphoric acid/50% ethanol solution, stained with Coomassie brilliant blue G250 and then washed twice in 50 mM ABC, twice in 50 mM ABC/50%ACN and once in 50 mM ABC. Gels bands (tissue lysate) were cut into ~ 1 mm^3^ cubes. The lane for each secretome was cut into 5 fractions and each fraction was cut into ~ 1 mm^3^ cubes. Gel cubes were reduced in 10 mM DTT/50 mM ABC for 1 h at 56 °C and alkylated in 50 mM iodoacetamide for 45 min at room temperature in the dark. After washing in 50 mM ABC/50% ACN, gel cubes were dried in vacuum centrifuge for 10 min at 50 °C. Following gel cube rehydration by trypsin solution (Promega, 6.25 ng/mL in 50 mM ABC), the gel cubes were covered with 50 mM ABC and incubated overnight at 25 °C. Peptides were isolated from the gel cubes with 5% FA/50% ACN (twice) and 1% formic acid (FA) (once). In a vacuum centrifuge at 60 °C the extracts were concentrated before LC–MS/MS after which volumes were adjusted to 50 μl with 0.05% FA into LC autosampler vials after filtering through a spin filter of 0.45 μm [18].

### NanoLC-MS/MS proteomic analysis

NanoLC-MS/MS measurement was performed as described previously [19]. Peptides were separated using an Ultimate 3000 nanoLC-MS/MS system (Dionex LC-Packings, Amsterdam, The Netherlands) equipped with a 40 cm × 75 μm ID fused silica column custom packed with 1.9 μm 120 Å ReproSil Pur C18 aqua (Dr Maisch GMBH, Ammerbuch-Entringen, Germany). After injection, peptides were trapped at 10 μl/min on a 10 mm × 100 μm ID trap column packed with 5 μm 120 Å ReproSil Pur C18 aqua in buffer A (buffer A: 0.1% formic acid in MQ; buffer B: 80% ACN + 0.1% formic acid in MQ) and separated at 300 nl/min in a 10–40% buffer B gradient in 90 min (130 min inject-to-inject) at 35 °C. Eluting peptides were ionized at a potential of + 2 kVa into a Q Exactive mass spectrometer (Thermo Fisher, Bremen, Germany). Intact peptide masses were measured at resolution 70.000 (at m/z 200) in the orbitrap using an AGC target value of 3 × 10^6^ charges. The top 10 peptide signals (charge-states 2^+^ and higher) were submitted to MS/MS In the HCD (higher-energy collision) cell (1.6 m/z isolation width, 25% normalized collision energy) using an AGC target value of 1 × 10^6^ charges an underfill ratio of 0.5% and a maxIT of 60 ms at resolution 17.500 (at m/z 200). Dynamic exclusion was applied with a repeat count of 1 and an exclusion time of 30 s. Each biological sample was injected twice and the LC–MS raw files for each sample were combined in the database search.

### Protein identification and database searching

OSCC MS/MS spectra were searched against the Swissprot human reference proteome FASTA file (canonical and isoforms) downloaded February 2019 (42,417 entries) using MaxQuant 1.6.4.0. HNSCC MS/MS spectra were searched against the Swissprot human reference proteome FASTA file (canonical and isoforms) downloaded January 2018 (42,258 entries) using MaxQuant 1.6.0.16 [20]. Two missed cleavages were allowed, and specificity of enzyme was set to trypsin. Methionine oxidation (Met, + 15.994915 Da) and N-terminal acetylation (N-terminal, + 42.010565 Da) was treated as variable modifications and Cysteine carboxamidomethylation (Cys, + 57.021464 Da) as fixed modification. Intact peptide ions were searched with a maximum mass deviation of 4.5 ppm and fragment ions with a maximum mass deviation of 20 ppm, (default MaxQuant settings). Using the target/decoy database search technique, peptide and protein identifications were filtered at an FDR of 1 percent. Proteins that could not be differentiated based on MS/MS spectra alone were grouped to protein groups (default MaxQuant settings).

### Protein quantitation and differential analysis

Spectral counting quantification of proteins was used, that is the sum of all MS/MS spectra for every detected protein [21]. Spectral counts for known proteins in a sample were normalized to the sum of spectral counts for that sample and subsequently multiplied by the mean of the sum for all samples. This procedure gives the relative spectral count contribution of a protein to all spectral counts in the sample. The normalized spectral counts were used to calculate the ratio of different biological sample groups. In that manner, we were able to correct for loading differences between samples. Differential analysis of proteins between samples was done using dedicated statistics by the beta-binominal test [22] using the R package countdata, which considers within- and between-sample variations. Cluster analyses of differentially expressed proteins were performed using hierarchical clustering in R, in which the protein abundances were normalized to zero mean and unit variance. For protein clustering, the Euclidean distance was used. The mass spectrometry proteomics data have been deposited to the Proteome Xchange Consortium via the PRIDE (http://proteomecentral.proteomexchange.org/) [23] partner repository with the dataset identifier PXD025701.

### Data mining, visualization & network analysis

The “protein–protein interaction network” analysis of up-regulated proteins in OSCC was performed using the STRING tool, version 10.5 [24] with medium stringency and default settings. The edges represent protein–protein interactions and nodes denote proteins based on different levels of evidence collected by String, and also visualized using MCL-cluster plugin of Cytoscape version 3.4.1 [25]. Gene ontology analysis was performed using the ClueGO app to get biological processes overrepresented and proteins involved (32) with cut-off 0.01 p-value. We used WebGestalt tool to do the GSEA. It was performed using a pre-ranked protein list as input. All proteins in the dataset were assigned a rank based on the differential expression analysis statistics (-log10 p-value multiplied with the sign of the fold-change) [26] and choose geneontology (biological process noRedundant) under functional database category to identify GO-biological-processes enriched for high and low expressed proteins. Expression plot was made in R. TFs which are responsible for regulating the expression of many of genes that mediate their biological activities like induction of cell-cycle arrest and apoptosis were identified using iRegulon [27]. The set criteria for motif enrichment analysis was as follows: FDR on motif similarity ≤ 0.001, identity between orthologous genes ≥ 0.0, and TF motifs with normalized enrichment score (NES) & gt; 3. The ranking preference for Motif collection was fixed to a putative regulatory region of 20 kb centered on TSS (7 species) and 10 K (9713 PWMs) was selected for the analysis. Thereafter, TF-target pairs were found based on the databases like TRANSFAC comprised in the iRegulon plugin. To predict non-classically and classically secreted proteins SignalP 5.0/SecretomeP 2.0 Server were used, whereby the signal peptide’s presence in the sequence of protein was used to categorize it as classically secreted and the threshold of NN score ≥ 0.6 and no existence of a signal peptide to categorize it as non-classically secreted proteins [28].

## Results and discussion

### Protein profiling of OSCC tissue vs NAT lysates

To identify altered biology and candidate biomarkers for OSCC, we performed mass spectrometry-based proteomics of cancer and adjacent normal tissues of 14 patients. For optimal label-free protein comparison, we ensured that equal protein amounts were loaded on the gels (Additional file 1: Fig. S2). In total, 5123 proteins were identified (Additional file 2: Table S1). About 3104 proteins were identified per sample. We used a paired beta-binomial test to identify the differentially expressed proteins. We used unadjusted p-values since the protein ranking does not change between adjusted and unadjusted values. To guard from multiple testing issues, we performed extra filtering using a fold change cutoff to identify candidates for further exploration. In addition, as the proteins do not act independently, the selected proteins were subsequently subjected to further network analysis. Finally, we used overlap analysis of multiple datasets to pinpoint biomarker candidates of high confidence and highest interest.

### Up-regulated protein networks associated with OSCC

**Fig. 1:**
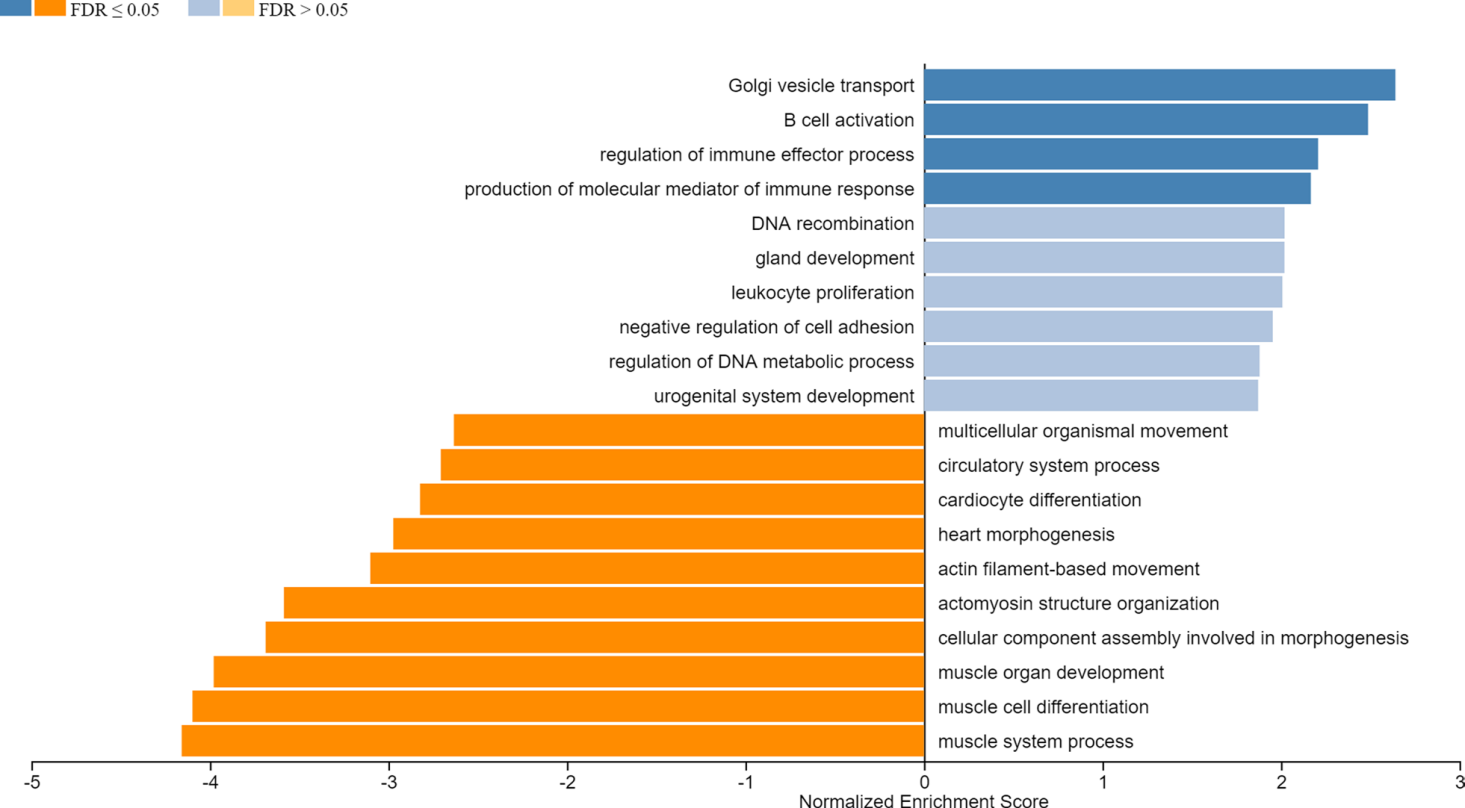
GSEA identified many deregulated biological processes, pathways up and down regulated shown in blue and orange color respectively. If FDR is smaller than or equal to 0.05, the colors of the bar are darker, while the color for categories with FDR larger than 0.05 is in a lighter shade

To study which proteins and pathways were associated with OSCC tissues, we performed differential analysis. A total of 205 upregulated proteins and 94 downregulated proteins (Additional file 3: Table S2) were significantly differentially expressed (p < 0.01, twofold up/down). First, we performed GSEA on the proteins identified in either the mucosa and OSCC samples to investigate the most prominent biological processes. This analysis revealed that secretion was the most significant pathway enriched in OSCC and myogenesis the pathway mostly reduced in tumor samples (or relatively enriched in mucosa samples) (Fig. 1). The latter likely indicates that the mucosa samples contained a relatively high number of muscle cells when compared to the OSCC samples. This relates to the different tissue architecture of either normal mucosa or tumor tissue and is hard to circumvent. For this reason we focused our analyses on the upregulated proteins, which are also easier to detect as biomarkers.

**Fig. 2:**
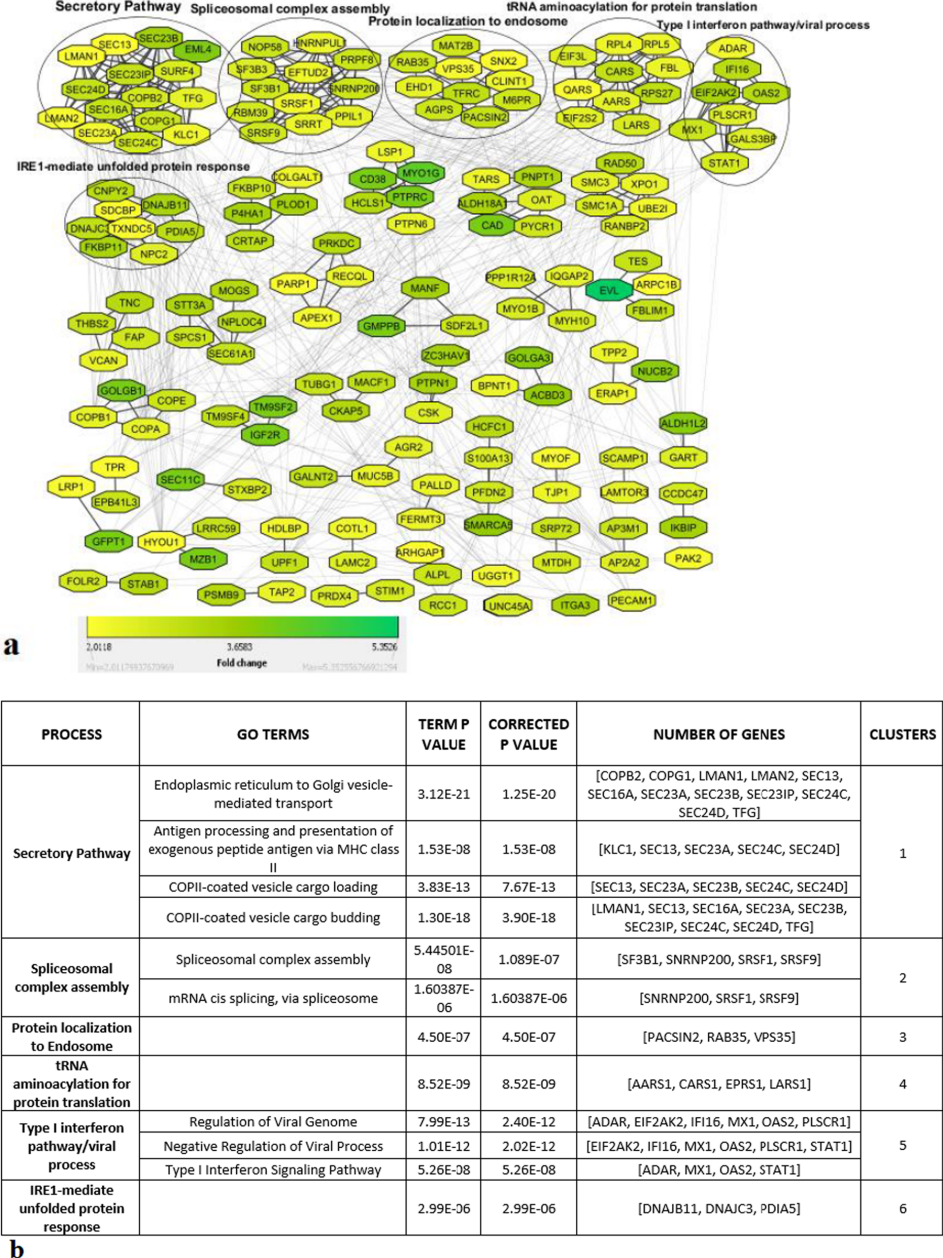
Up regulated biological processes associated with OSCC **a** String Interaction of 205 up regulated proteins at FC ˃ + 2 and P ˂0.01 in tumors as compared to NATs, Color intensity indicates fold changes according to the 2-group analysis. **b** Biological processes associated with the most populated protein clusters, analyzed using ClueGO

To gain further insight into the tumor biology, we generated a protein–protein interaction network for the 205 upregulated proteins in OSSC and found high connectivity with several tightly connected clusters (Fig. 2a, b, Additional file 4: Table S3). The 6 major clusters were enriched for proteins involved in secretory pathway (Cluster 1), spliceosomal complex assembly (Cluster 2), protein localization to endosome (Clusters 3), tRNA aminoacylation for protein translation (cluster 4), immunity (cluster 5) and protein biosynthesis (cluster 6).

Cancer cells are known to exhibit altered protein secretion to create a favorable tumor microenvironment [29]. The relative upregulation of the secretory pathway may indicate that OSCC tissues are secreting more proteins when compared to the paired normal tissues. These secreted proteins may also be suited to be exploited as non-invasive biomarkers, since they can be secreted into biofluids, including blood or saliva. In addition, the enrichment of biosynthesis pathways of aminoacyl tRNA may be explained by the active metabolism of cancer cells. Aminoacyl tRNA synthetases (ARSs) are vital enzymes that connect amino acids to their related tRNA. Beyond ARSs central role in protein translation, current studies have discovered others functions and expression levels may be associated to the prognosis of cancer [30, 31]. For example, in cancer cells stimulated by TNF, human lysyl-tRNA synthetase (KARS) is secreted that triggers proinflammatory signaling in immune cells [32].

### Tumor-promoting transcription factors in oral cancer

To predict the coordinated regulation of the proteins involved in the differential protein secretion and other biological processes associated with the highly connected protein clusters 1 to 6 (Fig. 2a), we analyzed whether specific transcription factors can be identified that regulate these proteins (Table 3). Transcription factor binding motifs were predicted in Cytoscape using the iRegulon plugin (version 1.3) [27]. We used proteins from cluster 1 to 6 as input proteins for motif and track search in the cis-regulatory control elements of genes of those proteins.

**Table 3:** List of Transcription factors responsible for different biological processes, and the proteins that are identified in the Secretomes of HNSCC cells

Serial. No	Most connected biological network Clusters	Target genes/Proteins	Transcription factors by iRegulon	Proteins secreted in cell line HNSCC secretomes
1	Secretory Pathway	KLC1, LMAN2, SEC24C, SEC23A, SEC23B, SEC16A, SEC13, TFG, SEC24D, EML4, COPB1, COPB2, GOLGB1, COPA, LMAN1, SEC23IP, SURF4, COPG1	Creb3L1	LMAN2, SEC24C, SEC23A, SEC23B, SEC13, TFG, SEC24D, EML4, COPB1, COPB2, GOLGB1, COPA, LMAN1, COPG1
2	Spliceosomal complex assembly	[SF3B1, SNRNP200, SRSF1, SRSF9]	ESSRA	[SF3B1, SNRNP200, SRSF1, SRSF9]
3	Protein localization to Endosome	[PACSIN2, RAB35, VPS35]	YY	[PACSIN2, VPS35]
4	tRNA aminoacylation for protein translation	[AARS1, CARS1, EPRS1, LARS1]	ELF2	[AARS1, CARS1, LARS1]
5	Type I interferon pathway/viral process	[ADAR, EIF2AK2, IFI16, MX1, OAS2, PLSCR1, STAT1]	STAT1	[ADAR, EIF2AK2, STAT1]
6	IRE1-mediate unfolded protein response	[DNAJB11, DNAJC3, PDIA5]	XBP1	[DNAJB11, DNAJC3]

We found that Creb3/Creb3L1, ESRRA, YY, ELF2, STAT and XBP1 were the central transcription factors that could explain the highly connected upregulated cluster-proteins 1 to 6 respectively as shown in Fig. 2a, b and Table 3. These TFs were previously reported to be upregulated in oral cancer [33–36] [37–40]. Targeting these TFs may alter the expression of many proteins. One of these TFs, STAT is known as the vital transcription factor regulating genes of the Type I interferon pathway/viral process. In our study, we found that expression of STAT1 was increased in OSCC tumor samples and secreted in HNSCC cell line secretome as well (Table 4).

**Table 4:** List of 25 OSCC tumor prediction biomarker candidate proteins (p < 0.01, FC > 2), and their detection in HNSCC cell line Secretomes and public saliva datasets

Accession number	Gene symbol	Razor + unique peptides count	Protein name	p-value	OSCC tumor vs NAT FCs	HNSCC cell line secretomes average counts	Frequency of detection in 03 saliva datasets	SecretomeP prediction	Subcellular localization
P49588	AARS	29	Alanine–tRNA ligase, cytoplasmic	0.001	2	78	3	NP	Cytosol
O15143	ARPC1B	18	Actin-related protein 2/3 complex subunit 1B	0.001	2	17	3	NP	Cytoskeleton
P27708	CAD	38	CAD protein;Glutamine-dependent carbamoyl-phosphate synthase;Aspartate carbamoyltransferase;Dihydroorotase	0.000	4	45	3	NP	Nucleus
P53618	COPB1	35	Coatomer subunit beta	0.001	2	22	3	NP	Cytosol
Q14019	COTL1	15	Coactosin-like protein	0.006	2	38	3	NCS	Endoplasmic reticulum
Q13217	DNAJC3	18	DnaJ homolog subfamily C member 3	0.000	3	18	3	CS	Nucleus
Q9HC35	EML4	21	Echinoderm microtubule-associated protein-like 4	0.000	4	11	3	NP	Cytoskeleton
Q9NZ08	ERAP1	18	Endoplasmic reticulum aminopeptidase 1	0.006	2	47	3	CS	Cytosol
Q06210-2	GFPT1	37	Glutamine–fructose-6-phosphate aminotransferase [isomerizing] 1	0.000	4	6	3	NP	Golgi apparatus
Q92896	GLG1	31	Golgi apparatus protein 1	0.001	2	34	3	CS	Nucleus
Q9Y4L1	HYOU1	37	Hypoxia up-regulated protein 1	0.002	2	37	3	CS	Plasma membrane
Q08380	LGALS3BP	11	Galectin-3-binding protein	0.001	3	269	3	CS	Plasma membrane
P49257	LMAN1	22	Protein ERGIC-53	0.003	2	7	3	CS	Plasma membrane
P61916	NPC2	9	Epididymal secretory protein E1	0.006	2	43	3	CS	extracellular
P80303-2	NUCB2	27	Nucleobindin-2;Nesfatin-1	0.000	4	9	3	CS	extracellular
Q02809	PLOD1	20	Procollagen-lysine,2-oxoglutarate 5-dioxygenase 1	0.001	3	74	3	CS	Nucleus
O14974	PPP1R12A	20	Protein phosphatase 1 regulatory subunit 12A	0.004	3	14	3	NP	Nucleus
Q13162	PRDX4	11	Peroxiredoxin-4	0.000	3	19	3	CS	Nucleus
P78527	PRKDC	110	DNA-dependent protein kinase catalytic subunit	0.000	3	67	3	NP	Nucleus
P46777	RPL5	17	60S ribosomal protein L5	0.003	2	38	3	NP	endoplasmic reticulum
Q9P2E9	RRBP1	71	Ribosome-binding protein 1	0.001	2	49	3	NP	Plasma membrane
Q14683	SMC1A	36	Structural maintenance of chromosomes protein 1A	0.002	3	28	3	NP	Nucleus
P42224	STAT1	38	Signal transducer and activator of transcription 1-alpha/beta	0.000	3	28	3	NP	Cytosol
P26639	TARS	29	Threonine–tRNA ligase, cytoplasmic	0.004	2	149	3	NP	Plasma membrane
Q07157-2	TJP1	27	Tight junction protein ZO-1	0.004	2	32	3	NP	Cytoskeleton

*CS* Classically secreted, *NCS* Non-classically secreted, *NP* Not predicted to be secreted

### OSCC tissue protein candidates predicted to be secreted

To further explore the process of secretion, we determined which of the deregulated tissue proteins are predicted to have a signal peptide using the SignalP database [28]. To gain more insight in the presumed subcellular localization of the proteins, the annotated subcellular localization was retrieved from the IPA database (Ingenuity® Systems www.ingenuity.com). Interestingly, when analyzing the 205 differentially expressed proteins (p < 0.01) of OSCC tumor tissue vs. paired normal adjacent tissue 38% of the proteins were predicted to be secreted, either via classical or non-classical secretion mechanisms (annotated in Additional file 11. Table S10). Overall, the proportion of proteins from the various subcellular origins was comparable between the OSCC tissues vs. NATs samples except for the proportion of cytoplasmic proteins̴ (~ 50%) that was much higher in OSCC tissues.

### Protein profiling of HNSCC cell line secretomes and comparison to OSCC tissue

Biological annotation revealed that the secretome pathway was highly enriched in OSCC tissues compared to normal mucosa. However, the differences in tissue architecture might have impacted these data. To further explore the potential of the secretome and annotate OSCC-associated proteins as candidate biofluid markers, we performed proteomics on the secretomes of HNSCC cell lines. We selected a diverse panel of 9 HPV-negative HNSCC cell lines to capture the complex tumor biology as much as possible. Cancer-associated proteins that are released may be more likely to be detectable in body fluids such as saliva [41]. Using an in-depth workflow based on gel fractionation coupled to nanoLC-MS/MS [42], we profiled the secretomes of HNSCC cell lines (Table 2). Prior to mass-spectrometry analysis, input quantities were checked by Coomassie blue-stained SDS-gel (Additional file 1: Fig. S3).

A total of 4472 cancer cell secretome proteins were identified (Additional file 5: Table S4), of which on average ~ 3500 proteins per sample. Of these, 1724 secretome proteins were identified (> 5 average counts) in all HNSCC secretomes (Additional file 6: Table S5). Overlap analysis of these robustly identified secretome proteins with the differential OSCC tissue proteins revealed 132 promising proteins as candidate non-invasive markers for HPV-negative HNSCCs. Underscoring their value as potential OSCC marker, 131 proteins were also cancer-associated in the large-scale proteomics analysis of OSCC tissue recently reported by Huang et al. 2021 (Additional file 7: Table S6) [7]. In our list of most overexpressed proteins in OSCC tissue or HNSCC secretomes, transferrin receptor (TFRC) was the most differentially expressed protein in tumors as compared to normal mucosa. Human Protein Atlas data expression of TFRC is high in HNSCC and it is also found abundant across different cancers, indicating that this is a common protein involved in multiple cancers. TFRC plays an essential role in the cellular uptake of iron. TFRC is related to lysosomes/endosomes, which can be secreted into saliva and blood (Additional file 1: Fig. S4a–c). In previous studies, TFRC expression rate in OSCC was found to be substantially higher than in dysplasia [43]. Interestingly, functional analysis in vitro and in vivo showed that an anti-TFRC antibody that blocks the interaction between transferrin and TFRC and consequently inhibits iron uptake, lead to the suppression of cell growth and induced apoptosis via iron deprivation [43]. Altogether the results of us and others suggest that TFRC may serve as a promising, cancer biology-linked biomarker for non-invasive diagnostics in OSCC.

**Fig. 3:**
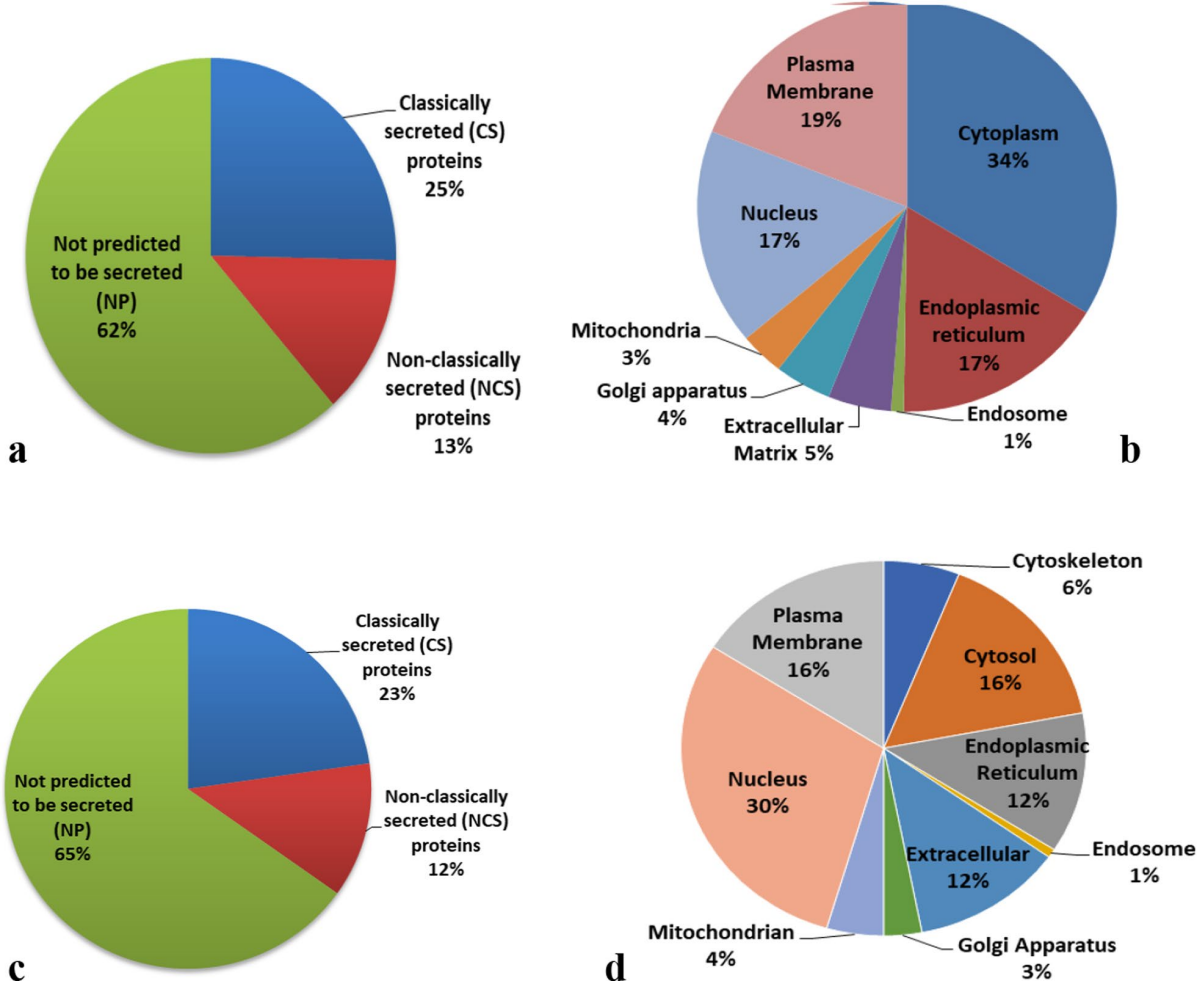
SecretomeP/SignalP analysis gives insight into differential biology of secretion and Proportion of protein subcellular localizations. **a** Percentage of classically secreted (CS) and non-classically secreted (NCS) proteins as well as those not predicted to be secreted (NP) by the SecretomeP algorithm in the OSCC tissue vs. NATs. **b** The pie charts show the percentage of different subcellular localizations of the total differential up regulated proteins in OSCC tissue vs. NATs samples. **c** Percentage of classically secreted (CS) and non-classically secreted (NCS) proteins as well as those not predicted to be secreted (NP) by the SecretomeP algorithm of 132 common identified proteins in the OSCC tissue vs. NATs and secretomes of HNSCC cell lines. **d** The pie charts show the percentage of different subcellular localizations of 132 common identified proteins

Strikingly*,* only 23% of the 132 overlapping OSCC tissue and HNSCC secretome candidates had a predicted signal peptide in their sequence (Fig. 3a–d, Additional file 8: Table S7). The remaining 16% of the proteins did not contain a classical signal peptide but were predicted to be secreted via non-classical routes, as predicted by the SecretomeP algorithm, which is based on other sequence-derived features and their subcellular localization [28]. Remarkably, among the proteins significantly more abundant in the HNSCC cell lines secretomes, the proportion of nuclear proteins was 30%. The biological background of this observation seems an enigma, but these secreted proteins might also serve as non-invasive biomarkers.

### Promising candidate biomarker for distinct clinical applications

**Fig. 4:**
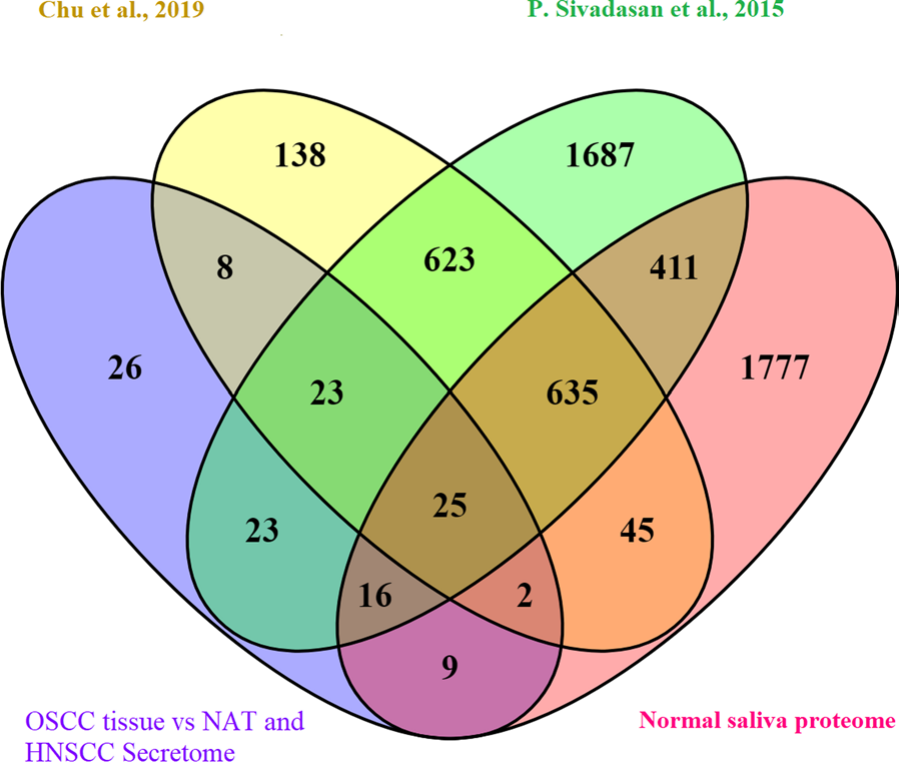
Venn diagram showing intersection of OSCC tissues vs. NATs and HNSCC cell line Secretomes and identified proteins in three published salivary datasets, i.e. salivary proteome healthy vs. OSCC dataset by Chu et al. [8], human salivary proteome [9] and Normal saliva proteome (https://salivaryproteome.nidcr.nih.gov/)

Non-invasive detection of OSCC might improve diagnosis of oral cancer. Therefore, we further annotated the 132 promising proteins from our OSCC tissue proteome (p < 0.01; > 2 upregulated) that are likely to be secreted (identified in all 9 cancer cell line secretomes with an average abundance of > 5 counts), for their potential use as non-invasive biomarker. To this end, we explored detectability in public proteomics datasets; salivary proteome data of normal mucosa vs. OSCC datasets [8], human salivary proteome [9] and the Normal Saliva Proteome database (https://salivaryproteome.nidcr.nih.gov/) (Additional file 9: Table S8). Importantly, 106 out of our 132 candidates (Fig. 4, Additional file 10: Table S9), have been identified as potential OSCC biofluid markers, with 25 top candidates detected in all 3 studies (Table 4). Therefore, these proteins may have the potential to be further exploited for developing a non-invasive biomarker test for the detection or prognosis of OSCC. Of these, THBS2, LGALS3BP and DNAJB11 were potentially useful salivary markers for the detection of OSCC as reported previously [41, 44–46].

## Conclusions

In conclusion, using comparative analysis of matched OSCC and paired normal mucosa tissues we identified proteins with different expression levels that form highly connected biological networks associated with a variety of distinct functional pathways, which may contribute to pathogenesis. Bioinformatics and overlap with cancer cell secretome and public saliva data identified an interesting set of OSCC proteins involved in secretory pathways and potential non-invasive biomarkers to explore for their ability to detect OSCC. Protein TFRC, the protein most abundantly expressed in OSCC cancer tissues, and identified in all cancer cell lines secretomes, is an especially promising candidate non-invasive biomarker that was previously not reported and merits further evaluation for its potential for the detection of OSCC.

## Supplementary Information

**Additional file 1: Figure S1. a** Moderately differentiated SCC **b** Well differentiated SCC. **Figure S2. **Similar protein loads of 14 paired tissue lysates (OSCC-tissues compared to normal adjacent tissue) on SDS-PAGE. **Figure S3.** Similar protein loads of 09 HNSCC cell lines lysates on SDS-PAGE. **Figure S4.** **a** Expression plot of TFRC (OSCC-tissues compared to normal adjacent tissue) by R. **b**, **c** Comparing the dataset of TFRC to the Human Protein Atlas.

**Additional file 2: Table S1. **List of 5123 identified proteins tumors compared to NAT of OSCC patients

**Additional file 3: Table S2. **List of differentially up and down regulated Proteins (P<0.01, 2-fold up/down) in tumors compared to NAT of OSCC patients.

**Additional file 4:** **Table S3.** String Interaction of 205 up regulated proteins at FC >+2 and P <0.01 in tumors as compared to NATs.

**Additional file 5: Table S4.** List of 4472 identified proteins secretomes of HNSCC cell lines.

**Additional file 6: Table S5.** 1724 secretome proteins were identified (˃5 average counts) in all HNSCC secretome.

**Additional file 7: Table S6. **Overlap analysis of these robustly identified secretome proteins with the differential OSCC tissue proteins revealed 132 promising OSCC proteins as candidate non-invasive markers. Underscoring their value as potential OSCC marker, 131 proteins were also cancer-associated in the large-scale proteomics analysis of OSCC tissue recently reported by Huang et al. [7].

**Additional file 8:** Table S7. 132 overlapping OSCC tissue and HNSCC secretome candidates had a predicted signal peptide in their sequence.

**Additional file 9: Table S8.**Public proteomics datasets; salivary proteome healthy vs. OSCC dataset by Chu et al. [8], human salivary proteome by Sivadasan et al. [9] and Normal Saliva Proteome database (https://salivaryproteome.nidcr.nih.gov/)

**Additional file 10: Table S9.**106 Common Proteins in "Tumor vs NATs OSCC and HNSCC Cell line Secrotome" vs "salivary proteome healthy vs. OSCC dataset by Chu et al. [8], human salivary proteome by Sivadasan et al. [9] and normal salivary proteome dataset (https://salivaryproteome.nidcr.nih.gov/)."

**Additional file 11: Table S10.** List of 205 differentially expressed up regulated proteins (P < 0.01) of OSCC tumor tissue vs. paired normal adjacent tissue predicted to be secreted, either via classical or non-classical secretion mechanisms and their subcellular localization

## Data Availability

The datasets generated or analyzed during the current study are available from the corresponding author on reasonable request.

## Abbreviations

NAT: Normal adjacent tissue
OSCC: Oral squamous cell carcinomas
HNSCC: Head and neck squamous cell carcinoma
LC–MS/MS: Liquid chromatography-mass spectrometry
GSEA: Gene set enrichment analysis
GO: Gene ontology
DEPs: Differentially expressed proteins
FCs: Fold changes
TFs: Transcription factors
