# Supplementary material for: Secreted protein markers in oral squamous cell carcinoma (OSCC)
Source: Clin Proteomics. 2022 Feb 8;19:4. doi: 10.1186/s12014-022-09341-5 (PMC8903575; doi:10.1186/s12014-022-09341-5)
Supplement: Supplementary file 1 — Additional file 1: Figure S1. a Moderately differentiated SCC b Well differentiated SCC. Figure S2. Similar protein loads of 14 paired tissue lysates (OSCC-tissues compared to normal adjacent tissue) on SDS-PAGE. Figure S3. Similar protein loads of 09 HNSCC cell lines lysates on SDS-PAGE. Figure S4. a Expression plot of TFRC (OSCC-tissues compared to normal adjacent tissue) by R. b, c Comparing the dataset of TFRC to the Human Protein Atlas. [file 12014_2022_9341_MOESM1_ESM.docx]

**SECRETED PROTEIN MARKERS IN ORAL SQUAMOUS CELL CARCINOMA (OSCC)**

Madiha Mumtaz^1^, Irene V Bijnsdorp^2^, Franziska Böttger^2^, Sander R. Piersma^2^, Thang V. Pham^2^, Samiullah^3^, Ruud H. Brakenhoff^4^, M Waheed Akhtar^1^, Connie R. Jimenez^2^

^1^School of Biological Sciences, University of the Punjab, Lahore-54590, Pakistan, ^2^Amsterdam UMC, OncoProteomics Laboratory, Department Medical Oncology, Location VUmc, Amsterdam, the Netherlands, ^3^Shalamar Medical and Dental College, Lahore, Pakistan, ^4^Amsterdam UMC, Vrije Universiteit Amsterdam, Otolaryngology / Head and Neck Surgery, Cancer Center Amsterdam, the Netherlands

**Additional file 1 FIGURES**

**S-1**

a.
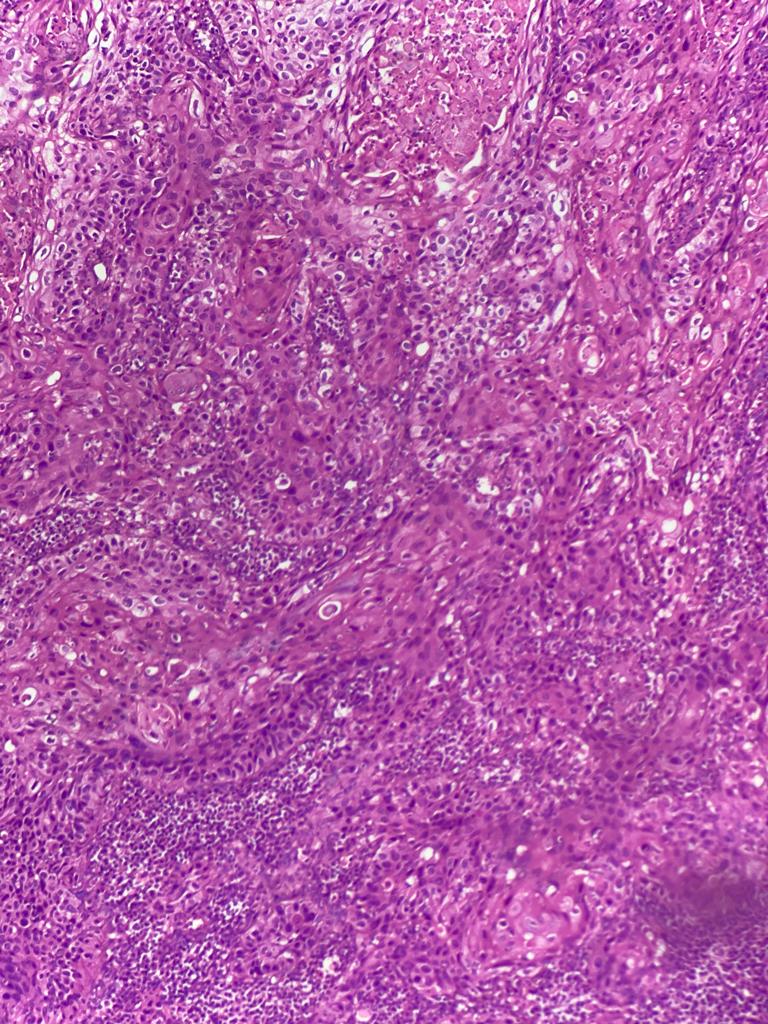
 b.
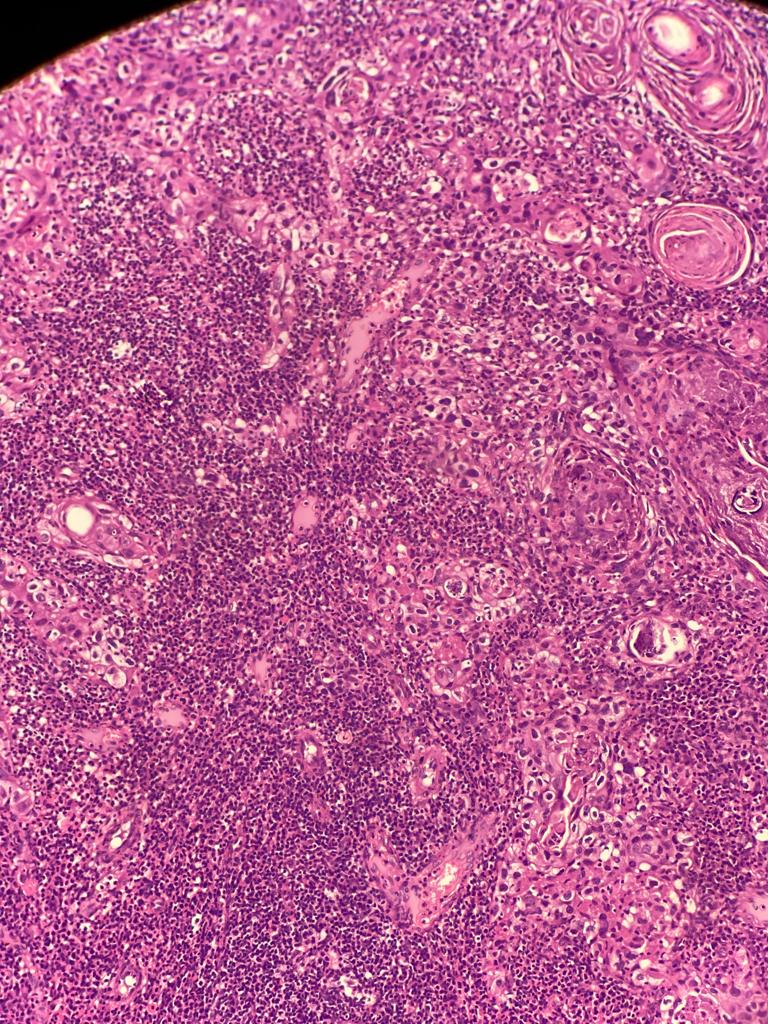


**Additional file 1: Figure S1. a.** Moderately differentiated SCC **b.** Well differentiated SCC

**S-2**


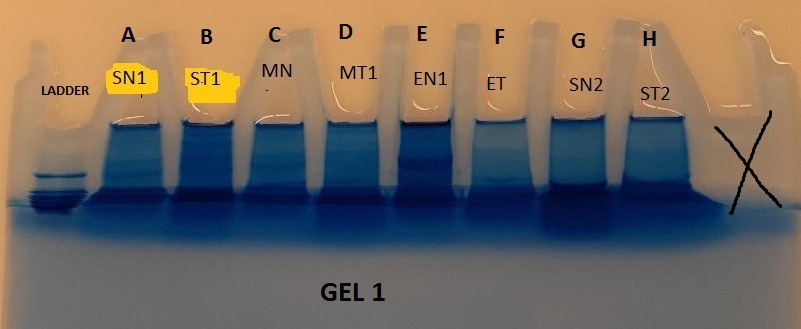


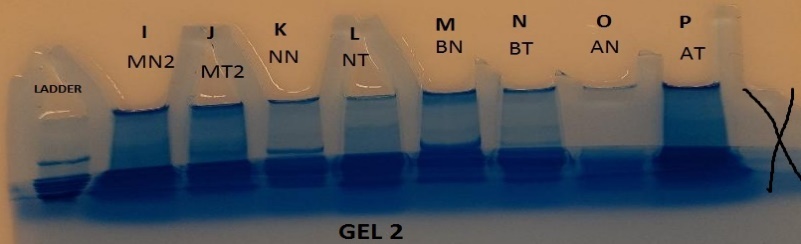


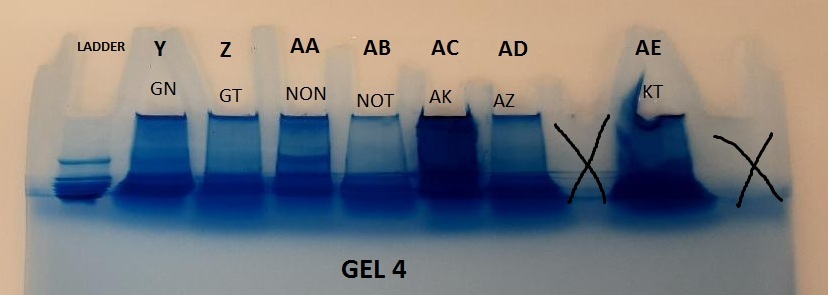

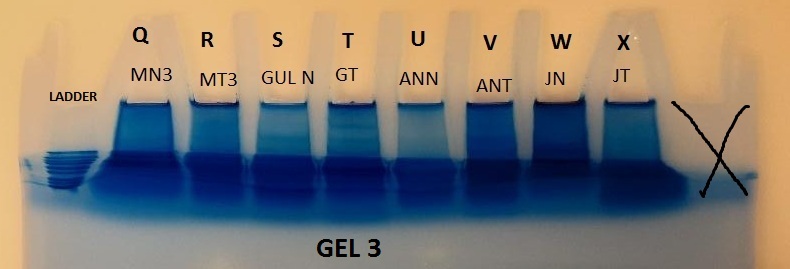


**Additional file 1: Figure S2.** Similar protein loads of 14 paired tissue lysates (OSCC-tissues compared to normal adjacent tissue) on SDS-PAGE

**S-3**


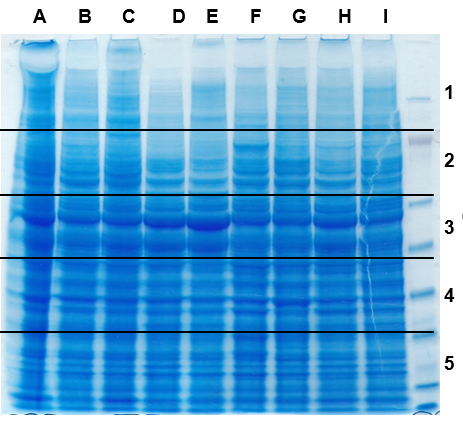


**Additional file 1: Figure S3.** Similar protein loads of 09 HNSCC cell lines lysates on SDS-PAGE

**S-4**

**a b**


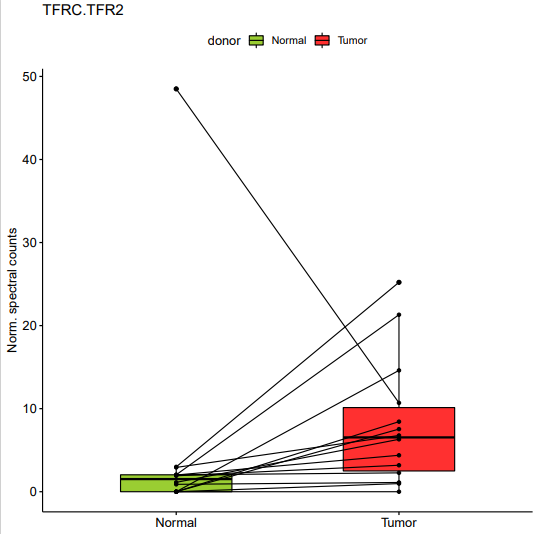

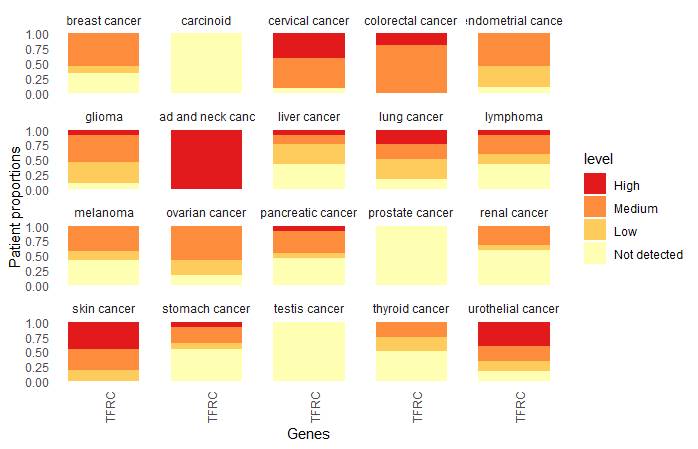


**c**
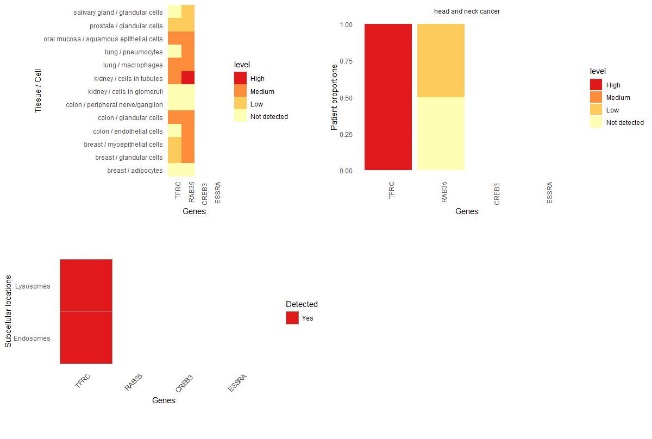


**Additional file 1: Figure S4.** **a** Expression plot of TFRC (OSCC-tissues compared to normal adjacent tissue) by R. **b and c.** Comparing the dataset of TFRC to the Human Protein Atlas
